# The Metabolic and Antioxidant Activity Profiles of Aged Greek Grape Marc Spirits

**DOI:** 10.3390/foods13111664

**Published:** 2024-05-26

**Authors:** Charalambos Fotakis, Vasiliki Andreou, Dionysios C. Christodouleas, Maria Zervou

**Affiliations:** 1 Institute of Chemical Biology, National Hellenic Research Foundation, 48 Vas. Constantinou Ave., 11635 Athens, Greece; bfotakis@yahoo.com (C.F.); vicky.x.a@hotmail.com (V.A.); 2 Department of Chemistry, University of Massachusetts Lowell, Lowell, MA 01854, USA; dionysios_christodouleas@uml.edu

**Keywords:** NMR-metabolomics, tsipouro, multivariate data analysis, spectrophotometry, barrel aging

## Abstract

In the last decade, “expressions” of grape marc spirits aged in wooden barrels of characteristic amber color and complex sensory attributes have been introduced. Yet studies on constituents migrating from the barrel to the beverage are scarce, and their metabolic profile remains unexplored. Furthermore, the literature on the assessment of their antioxidant activity is limited. NMR metabolomics and spectrophotometry have been implemented in 38 samples to elucidate the impact of the aging procedure on the metabolites’ composition and establish whether these beverages exhibit antioxidant activity. Provenance was related to fusel alcohols, esters, acetaldehyde, methanol, saccharides, and 2-phenylethanol, while ethyl acetate and ethyl lactate contributed to discriminating samples of the same winery. Identified metabolites such as vanillin, syringaldehyde, and sinapaldehyde were related to the aging procedure. The maturation in the barrel was also associated with an increase in xylose, glucose, fructose, and arabinose. The antioxidant potential of the aged Greek grape marc spirits resulting from their maturation in oak barrels was highlighted. The metabolic profiling and antioxidant potential of aged Greek grape marc spirits were assessed for the first time. Finally, the enrichment of the aromatic region was noted with the presence of metabolites with a furanic and phenolic ring derived, respectively, from the polysaccharides’ degradation or the thermal decomposition of lignin.

## 1. Introduction

*Tsipouro* and *Tsikoudia* are the Greek grape marc spirits, widely recognized as colorless liquids with intense organoleptic properties and high alcoholic strength (minimum 37.5% by volume). 

In the last decade, new “expressions” of these traditional products have emerged with a characteristic amber color and complex sensory attributes. These are aged Greek grape marc spirits that have matured in wooden barrels for at least a year. In fact, before bottling occurs, it is a common practice for beverages to undergo “maturation” in order to achieve consistent aging [1]. This is a core aspect in the manufacture of these beverages, as it is both a timely and costly procedure, but essential to a beverage’s unique flavor, depending on the type of alcohol and wood matrix utilized [2]. During aging, the contact with wood confers to the beverage unique physicochemical and sensory characteristics. A plethora of phenomena contribute to the distinct profile of an aged beverage, i.e., the migration of wood compounds towards the beverage, and the entrance of oxygen in the barrel by diffusion or by micro-oxygenation resulting in oxidation and nonoxidative processes [3]. Especially, phenolics transferred from the barrel into the beverage play a key role in the color, aroma, flavor, and mouthfeel sensations of these beverages and may thus be perceived and even sought after by consumers [3].

The aging of an alcoholic spirit is a practice entwined with the production of whiskey. In this direction, grape marc spirits producers in Greece tend to try and age their tsipouro or tsikoudia in an effort to add enhanced flavor and even confer nutritional value.

Evidently, the aging process parameter is a critical factor for the metabolite composition of a beverage, such as wine or grape marc spirit. Moreover, very few studies have investigated the stages of the aging process of grape marc spirits. For instance, researchers attempted to determine the optimum conditions in the extraction of volatile compounds and phenolic substances from oak chips to the Orujo spirit [4]. Additionally, the golden rum spirit was put under the scope of ^1^H NMR spectroscopy to elucidate the effect of fermentation barrel, raw material, distillation method, and aging [5].

In particular, the scientific interest in grape marc spirits has mainly focused on their major volatile compounds with the implementation of Headspace Solid-Phase Microextraction Gas Chromatography–Mass Spectrometer (HS-SPME-GC-MS). Such a study associated volatile metabolites of tsipouro to geographical areas and production styles [6], while another determined the volatile composition of 39 samples of grape marc spirits to identify terroir denomination [7]. Our laboratory has contributed significantly to assessing the Greek grape marc spirit metabolome. Previously, with the application of NMR metabolomics, we investigated Greek grape marc spirits of indigenous and international varieties in order to establish their authenticity and assess their metabolic profile in relation to origin, variety, and vintage [8,9].

Albeit genetic (cultivars) [10], viticultural, or oenological practices’ [11] environmental impact (climate, soil, and cultural practices together referred to as “terroir”) [12], as well as the vintage effect, influence the metabolic profile of grape-derived products, the mosaic of factors and their interaction impede the assessment of quality and authenticity for such everyday commodities [13,14,15]. Therefore, the application of state-of-the-art methodologies is necessary in order to access the integrated metabolic profile of a grape-derived product. Specifically, the use of NMR metabolomics facilitates the acquisition of a holistic perspective in monitoring their consistency and framing metabolite perturbations [13], but although in recent years the studies assessing the metabolic profile of wines have increased, the literature on grape marc spirits remains scarce [15].

On these grounds, the current study aspires to accomplish two main objectives. First, to delineate the impact of the aging process on their metabolic composition, and second, to assess the antioxidant profiles of Greek grape marc spirits. Towards this aim, NMR-based metabolomics combined with multivariate statistics monitored the metabolic signature and identified patterns related to the aging process. Moreover, an array of complementary spectrophotometric methods was implemented to provide a more comprehensive evaluation of the antioxidant activity profile of the samples. Thus, free radical scavenging activity was evaluated by DPPH and ABTS•+ methods and the reducing capacity was estimated by FRAP and CUPRAC methods, while the total phenolic content via Folin–Ciocalteu and the o-diphenolic content were also evaluated. The collected samples stem from the northern continental Thessaly (central Greece) District and the Peloponnesus Peninsula (Southeastern Greece). The sample pool was extended to a host of grape varieties in order to discern the influence of the genotypic factor and the degree of origin influence when the aging process parameter is also incorporated.

## 2. Materials and Methods

### 2.1. Instruments

NMR spectra were acquired on a Varian-600 MHz NMR spectrometer equipped with a ^1^H{^13^C/^15^N} 5mm PFG Automatable Triple Resonance probe at 25 °C.

Measurements of the antioxidant capacity were carried out on a JASCO V-500 UV-Vis spectrophotometer.

### 2.2. Samples and Chemicals

The sample set included 38 grape marc spirits and was collected from one cooperative association and one winery. Specifically, 26 tsipouro samples from the viticulture area of Nemea in the region of Peloponnesus, southern Greece, included 6 fresh and 6 aged samples in French oak barrels for one year (100% Agiorgitiko, 2019 vintage year) and 7 fresh and 7 aged samples in French oak barrels for two years (25% Moschofilero, 25% Chardonnay, 50% Muscat Hamburg, 2019 vintage year). Moreover, 12 tsipouro samples gathered from the viticulture area of Tyrnavos in the Thessalian Plain, central Greece, encompassed 6 fresh and 6 aged samples in French oak barrels for one year (100% Muscat Blanc, 2019 vintage year). The samples were analyzed within one week of their supply. The bottles prior to the analysis were stored in a dry, clean, well-ventilated area at a room temperature of 25 °C.

Ethanol absolute and sodium molybdate 2-hydrate were purchased from Panreac, D_2_O (99.9%), DSS (97%), gallic acid and caffeic acid were obtained from Sigma, sodium carbonate anhydrous, and neocuproine hemihydrate were purchased from Fluka, ethanol 95%, Folin–Ciocalteau reagent, sodium hydroxide, acetic acid glacial 100% anhydrous, cupric chloride, iron(III) chloride hexahydrate, ammonium acetate Cryst. Extrapure, potassium persulfate, 2,2-azino-bis-3-ethylbenzothiazoline-6-sulphonic acid (ABTS), diammonium salt, and sodium acetate trihydrate were obtained from Merck, 2,4,6-Tri-(2-pyridyl)-1,3,5-triazine (TPTZ) 98% from Alfa Aesar, 2,2-Diphenyl-1-Picrylhydrazyl (DPPH) 95% from Aldrich-Chemie, hydrochloric acid S.G. 1.18 36% from Fisher Scientific, sodium acetate anhydrous G.R. from Lach-Ner, and deionized water.

### 2.3. NMR-Based Metabolic Fingerprinting

Samples for the NMR analysis were prepared by dissolving 200 μL of grape mark spirit in 200 μL of D_2_O, mixed with 140 μL oxalate buffer (400 mM, pH 4.0) and 60 μL DSS (5 mM).

^1^H NMR spectra were acquired by the use of WET1D sequence with selective suppression of the ethanol resonances as well as of the water residual signal and applying a decoupling scheme to eliminate the ^13^C-satelites of the suppressed peaks.

Spectra were acquired with 256 transients using 72 K data points across a spectral width of 9615.4 Hz. A relaxation delay of 1.5 s, an acquisition time of 4.00 s, and a flip angle of 45° were applied to ensure full recovery of the bulk magnetization along the *z*-axis. The applied experimental protocol enables the quantitative analysis of the contained metabolites in the intact samples to bypass the dynamic range limitations due to the increased ethanolic matrix. This is extensively documented in our previous work [14,15].

Metabolites annotation was assisted by 2D NMR spectroscopy (COSY, zTOCSY, HMBC, HSQC), and Chenomx database (NMR Suite 7.0) 2D NMR spectra were recorded with routine Vnmr pulse sequences as previously described [15].

#### 2.3.1. Postprocessing of NMR Data

^1^H-NMR spectra process included thorough phase and baseline correction, alignment, and reduction into spectral buckets of 0.0001 ppm by MestreNova (v.10.1) software (Santiago de Compostela, Spain). The following regions were removed: D_2_O (4.7–5.0 ppm), ethanol resonance peaks (1.15–1.20 ppm and 3.59–3.72 ppm), and the region of buffer (1.9–2.0 ppm). The spectra were aligned, normalized to the standardized area of DSS, and converted to ASCII format using the Mnova processing template.

#### 2.3.2. Multivariate Data Analysis

SIMCA-P (version 14.0, Umetrics, Umeå, Sweden) was applied. The matrix of the processed spectral data sets was imported into the SIMCA-P version 14.0 (Umetrics, Umeå, Sweden) for statistical analysis.

A PCA model was extracted to visualize any clustering among the observations (samples). The spectral data were mean-centered with Pareto scaling (Par), and the model was extracted at a confidence level of 95%.

Then, classification analysis ensued by further subjecting the data set to orthogonal projections to latent structures–discriminant analysis (OPLS-DA). This regression model enables the extraction of a single first component, a predictor for the class that associates with the between-groups variation, while the other extracted components describe the remaining variation orthogonal to the first predictive component and link to the within-groups variation [16,17]. The extracted OPLS-DA models were mean-centered and Pareto-scaled (Par) at a confidence level of 95%.

**Important feature selection**: Contribution plots created from the PCA score plot revealed the spectral areas mostly affecting the samples’ differentiation between the selected groups. Further, S-line plots extracted for the OPLS-DA two classes’ models enabled the visualization of the predictive component loading with the color coding highlighting the significantly altered metabolites that influence the separation of the groups.

**Model evaluation**: The quality of models (PCA/OPLS-DA) was described by the goodness-of-fit R^2^ (0 ≤ R^2^ ≤ 1) and the overall predictive ability Q^2^ (0 ≤ Q^2^ ≤ 1) values [17]. In particular, all models demonstrated high statistical values (R^2^ > 0.79 and cumulative Q^2^ ≥ 0.7), with the difference between the goodness-of-fit and the predictive ability always remaining lower than 0.2 (R^2^X(cum) − Q^2^(cum) < 0.2), indicative of a robust model with enhanced predictive response.

OPLS-DA regression models were validated through cross-validation analysis of variance (CV-ANOVA), with a *p*-value < 0.05, and response permutation testing (999 permutations) to check the validity of prediction and ensure the avoidance of overfitting. Moreover, the predictive accuracy of the OPLS-DA models was assessed through ROC analysis and calculation of the area under ROC curve (AUROC). A perfect discrimination corresponds to an AUROC equal to 1.

All these validation steps concur with the correct assignment of all objects in each class for all the OPLS-DA models [17,18].

Specifically, the performance of all OPLS-DA models was validated via permutation testing and receiver operator characteristic (ROC) curve, as displayed in Supplementary Figures S2–S5.

### 2.4. Antioxidant Activity Profile

In each spectrophotometric assay, three measurements for each sample were acquired.

#### 2.4.1. Determination of Total Phenolic Content Using the Folin–Ciocalteau Assay

The total phenolic content of each sample was determined by applying a modified method of Folin–Ciocalteu’s colorimetric assay according to Andreou et al. [19]. The total phenolic content was expressed as mg gallic acid equivalents (GAE) per L of grape marc spirit, using a standard curve with 50–650 mg/L gallic acid (*y* = 0.0017*x* − 0.0395, *R*^2^ = 0.9920).

#### 2.4.2. Assessment of Total O-Diphenolic Content

The methodology described in [20] was followed. The absorbance was measured at 370 nm. As a standard substance aqueous solution of caffeic acid was used. The results are expressed as caffeic acid equivalents (CAE) per liter of grape marc spirit.

#### 2.4.3. Assessment of Free Radical Scavenging Activity Using DPPH Assay

The antiradical activity of grape marc spirit samples was determined according to the method described by Tafulo et al. [21]. The absorbance was measured at 525 nm. Results are expressed as GAE per liter of grape marc spirit.

#### 2.4.4. Assessment of Free Radical Scavenging Activity Using ABTS•+ Assay

The methodology described in [20] was followed. The absorbance was measured at 734 nm. Results are expressed as GAE per liter of grape marc spirit.

#### 2.4.5. Assessment of Reductive Capacity Using FRAP Assay

The methodology described in [20] was followed. The absorbance of the mixture was measured at 593 nm. Results are expressed GAE per liter of grape marc spirit.

#### 2.4.6. Assessment of Reductive Capacity Using CUPRAC Assay

The methodology described in [21] was followed. The absorbance was measured at 450nm. Results are expressed as GAE per liter of grape marc spirit.

A detailed description of the steps followed in each assay is presented in the Supplementary Material, S6.

### 2.5. Statistical Analysis

The values were averaged and reported along with their standard error of the mean (S.E.M.). The Kolmogorov–Smirnov tests were applied to the results of the six spectrophotometric assays to examine normality. A normal distribution was detected, and a Pearson correlation was performed to extract the correlation coefficients r. A two-tailed unpaired *t*-test was applied for the comparison of all data concerning total phenolic content, o-diphenol content, and antioxidant and antiradical activities. These calculations were performed with the IBM SPSS Statistics 23 software.

## 3. Results and Discussion

Our endeavor in investigating aged Greek grape marc spirits of indigenous and international varieties, stemming from the major vine growing regions of Greece, is presented. This is based on two approaches: first, the application of NMR metabolomics, and second, the employment of spectrophotometry.

### 3.1. H NMR Metabolic Profile

A grape marc spirit is characterized as “aged” when it remains in oak barrels for at least six months. The mechanisms involved during its presence in the barrel are extremely complex and contribute to a large extent to the flavor development of these beverages. A series of stabilizing reactions occur in the oak barrel that affect color, phenolic composition, and the enhancement of aromatic profile. None of these reactions can occur in bottles or stainless tanks, because these are sealed containers and thus do not interact with the beverage [22]. During the aging of wines and spirits in oak barrels, a gradual solubilization of ellagic tannins occurs and oxidative processes gradually transform them into polymerized and colored polyphenolic molecules. Thus, they affect the color, astringency, and bitterness of the beverage [12,13].

In our previous study [8] on Greek grape marc spirits, we accomplished an assessment of the metabolic profile of fresh samples. An overlay of spectra from fresh and aged spirits is displayed in Supplementary Figure S1, with annotations on metabolites contributing to the results’ interpretation. First of all, the presence of proto-Quercitol, a cyclic polyalcohol, is of interest (Supplementary data, Table S1). This metabolite is a deoxyinositol characteristic of all Quercus species and has been proposed as an authentic indicator of wine aging in oak barrels [22].

The composition of sugars has been reported as an indicator of differentiation of Brandy Jerez in relation to its aging [23]. In our samples, the sugar region exhibits differences with saccharides (xylose, glucose, fructose, arabinose) that are increased in the aged spirit.

Finally, the aromatic region has been enriched with metabolites with a furanic and phenolic ring that may have antioxidant capacity. Phenolic acids such as syringic and gallic acids, originate from aging in oak barrels. Terpenes are hydrolyzable tannins that are released in the grape marc spirit and facilitate oxidative reactions, such as ester formation between alcohols and acids. Tannins contribute to the astringency of the grape marc spirit and when oxidized impart color and release aromatic compounds, such as gallic acid and ellagic acid. Furans can be formed in three ways: pyrolysis of carbohydrates, dehydration of sugars through the Maillard reaction, and caramelization. Their concentration is also influenced by the aging process [24].

The main phenolic aldehydes identified are vanillin, syringaldehyde, and sinapaldehyde. They derive from lignin fragments as a result of hydrolysis, pyrolysis, and oxidation reactions and contribute to the organoleptic characteristics of the beverage. The furanic and phenolic compounds identified in aged Greek grape marc spirits are displayed in Supplementary data, Table S1.

The observed prominent enrichment with aromatic compounds in Greek grape mark spirits as a result of the aging process is indeed significant. These aromatic compounds not only enhance the flavor and aroma profile of the spirits but also contribute to potential health benefits associated with moderate consumption.

### 3.2. Chemometrics Analysis on the NMR Metabolomics Data

**Figure 1:**
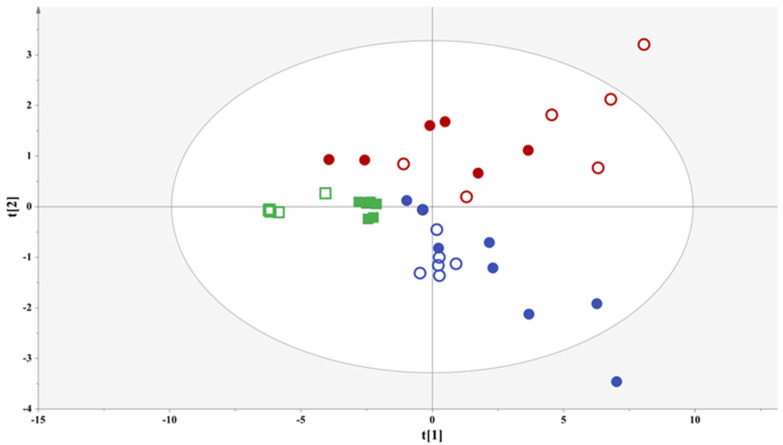
PCA model of the entire sample set, Par scaling, A = 2, N = 38, R^2^x(cum) = 0.79, Q^2^ (cum) = 0.70, (squares = samples from Thessaly District, circles = samples from Peloponnesus Peninsula, empty symbol = fresh grape marc spirit, full symbol = aged grape marc spirit). The different colors encode varietal information (red = 100% Agiorgitiko, blue = 25% Moschofilero, 25% Chardonnay, 50% Muscat Hamburg, green = 100% Muscat Blanc).

A PCA model was extracted incorporating all the samples, with two components explaining 79% of the data variation (PC1 explaining 65% of the data variation and PC2 explaining 14%), featuring both high goodness of fit (R^2^x(cum) = 0.79) and high predictive ability (Q^2^ (cum) = 0.70) (Figure 1). In fact, the PCA model reveals the contribution of three factors in the classification of the samples: the geographic origin, the grape variety, and the aging process.

#### 3.2.1. Comparing Samples of Different Geographic Origins

Interestingly, the first and notably most significant factor in classifying the samples is geographical origin, since its impact is evident along the first component. Specifically, the samples from Thessaly are located in the second and third quadrants, while the spirits from the Peloponnesus region are mainly located in the first and fourth quadrants.

**Figure 2:**
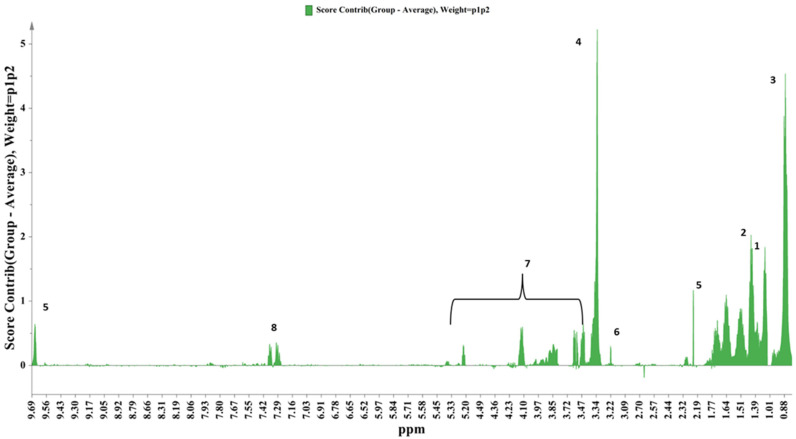
Contribution plot of samples stemming from Thessaly as compared with the rest of the samples stemming from Peloponnesus (1: ethyl acetate, 2: ethyl lactate, 3: fusel alcohols, 4: methanol 5: acetaldehyde 6: methyl acetate, 7: saccharides 8: 2-phenyl ethanol, 9: succinic acid).

The observed geographical differentiation trend can be interpreted by the contribution plot in Figure 2, in which the region discriminant metabolites are depicted. This plot highlights the spectral regions that bear class discriminant information regarding the geographical signature, which could reflect the environmental and viticultural practices’ influence. Characteristic metabolites for the samples stemming from Thessaly are fusel alcohols, ethyl acetate, ethyl lactate, acetaldehyde, methyl acetate, methanol, saccharides, and 2-phenylethanol.

Fusel alcohols have previously been linked to a “terroir” that may elevate the nitrogen sources in the grape and increase the levels of the high alcohols’ precursors, the amino acids [25].

NMR studies have ascribed a genotypic attribute and a marker of environmental influence to 2-phenyl ethanol [26].

Increased methanol and acetaldehyde content can be explained as metabolite variations due to differences in the yeasts used and microbial growth during alcoholic and malolactic fermentations between the two wineries.

Saccharides are primary metabolites that are strongly influenced by environmental factors, as mirrored in studies referring to grape vines of different geographical origins [27].

Differences in the concentrations of ethyl and methyl acetate may even be attributed to differences in aerobic conditions during fermentation and probable acetic bacterial spoilage or incorrect separation of the distillation fractions [28].

A characteristic metabolite for the Peloponnesus samples is succinic acid. This metabolite’s levels are mainly affected by the fermentation behaviors of wine yeast or lactic acid bacteria [29] and have even been related to factors such as the vintage and the grape cultivar [29,30].

#### 3.2.2. Comparing Samples from the Same Winery

PCA probed a second factor in the classification of samples, the grape variety; since, in Figure 1, clear groupings of the samples were evident based on the grape cultivar. In alignment with this observation, we further compared samples from the same producer in the Peloponnesus region, i.e., Agiorgitiko and the blend containing the Moschofilero, Moscato, and Chardonnay varieties, to efface the influence of the other factors contributing to the metabolites’ variation.

**Figure 3:**
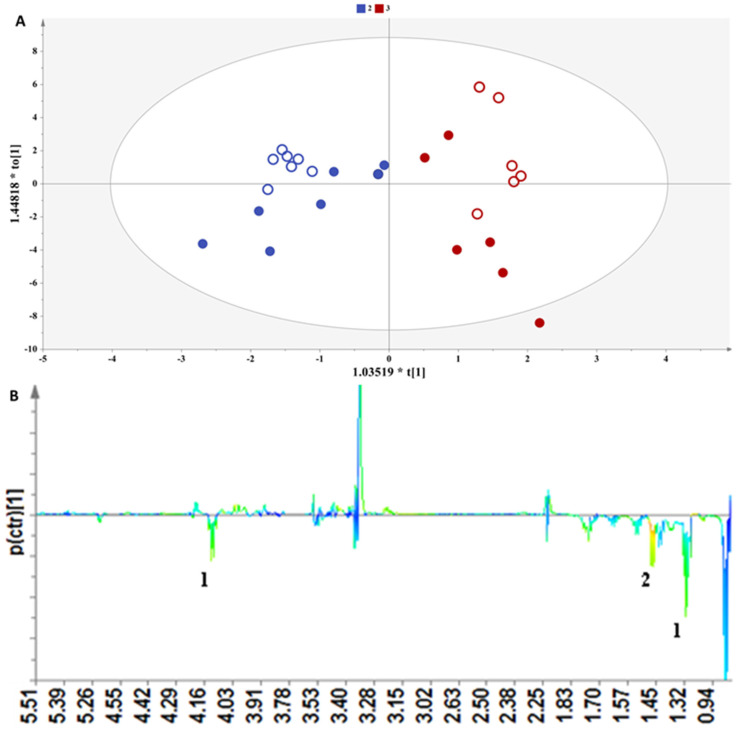
(**A**) OPLS-DA model applied to Peloponnesus samples with grape variety as class information. Par scaling, A = 1 + 1, N = 26, R^2^x(cum) = 0.63, R^2^y(cum) = 0.86, Q^2^ (cum) = 0.78, *p*-value = 6.29251 × 10^−7^. Samples representation coding: empty symbol = fresh grape marc spirit, full symbol = aged grape marc spirit, red = 100% Agiorgitiko, blue = 25% Moschofilero, 25% Chardonnay, 50% Muscat Hamburg, (**B**) S-line plot (1: ethyl acetate, 2: ethyl lactate).

In Figure 3A, a clear discrimination along the first principal component between the samples of Agiorgitiko and the blend containing the Moschofilero, Moscato, and Chardonnay varieties is observed. The corresponding S-line plot associates the ester fingerprint to this differentiation present with an enhanced concentration to the blended samples (Figure 3B). The content of ethyl acetate and ethyl lactate strongly affects the organoleptic characteristics of a beverage and depends on the lipid metabolism and acetyl CoA chemical esterification of alcohols and acids [29].

#### 3.2.3. The Impact of Aging Procedure

The aging procedure as a third factor was also hinted to affect the localization of the samples, in accordance with the formation of distinct subgroups in the PCA model (Figure 1). In this context, fresh and aged samples of common varietal composition were compared in order to determine metabolites evident of aging and monitor the transfer of substances from the oak barrel to the grape marc spirit. For this purpose, the data sets were further subjected to OPLS-DA in order to increase the quality of the classification model.

**Figure 4:**
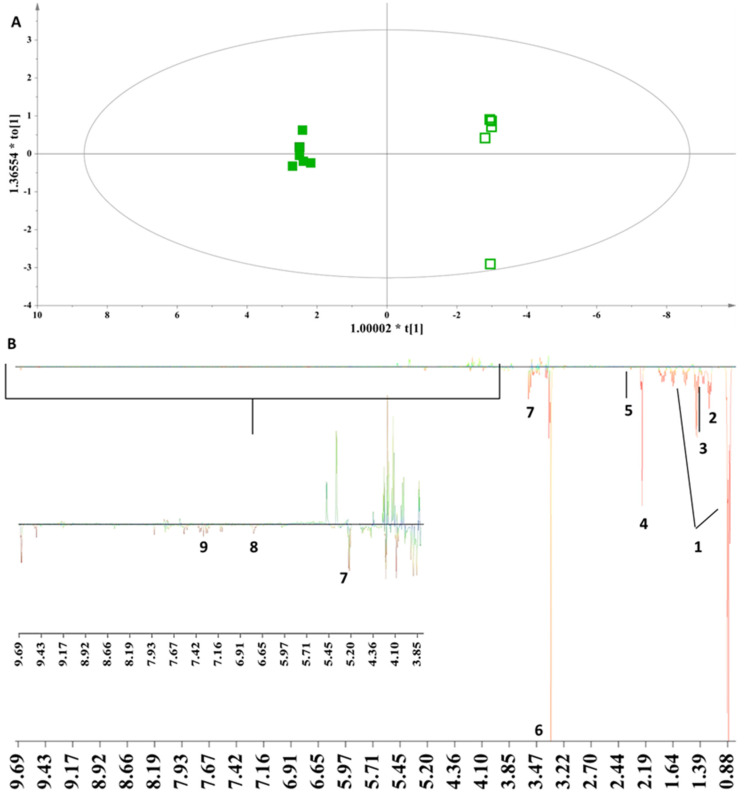
(**A**) OPLS-DA model on the single varietal (100% Muscat Blanc) samples from Thessaly Par scaling, A = 1 + 1, N = 12, R^2^x(cum) = 0.86, R^2^y(cum) = 0.99, Q^2^ (cum) = 0.97, *p*-value = 9.27094 × 10^−5^. Samples representation coding: empty symbol = fresh grape marc spirit, full symbol = aged grape marc spirit in French oak barrels for one year. (**B**) S-line plot (1: fusel alcohols, 2: ethyl acetate, 3: lactic acid, 4: acetaldehyde, 5: pyruvic acid, 6: methanol, 7: saccharides (glucose, fructose, xylose, and arabinose), 8: gallic acid, 9: phenolic and/or furanic metabolites).

**Grape Marc Spirits from Thessaly:** In Figure 4, an OPLS-DA model is extracted with the grape marc spirits from Thessaly, depicting a clear discrimination between aged and fresh samples along the first principal component. This is interpreted and assigned to metabolites with the S-line plot in Figure 4B. Thus, a more complex metabolic bouquet is revealed for the aged samples, as they are characterized by increased concentrations of fusel alcohols, acetaldehyde, ethyl acetate, lactic acid, pyruvic acid, saccharides (glucose, fructose, xylose, and arabinose), methanol, and gallic acid, as well as aromatic metabolites with a phenolic and/or furan ring.

In general, aging in wooden barrels mitigates volatility and increases the complexity of the sensory characteristics of the spirit, which is evident in the framed metabolite changes in Figure 4B with the increased content of fusel alcohols and ethyl acetate. As a result, the significant modification of the organoleptic characteristics of the grape marc spirit occurs (flavor, aroma, color) by compounds that have been transferred from the charred surface of the barrel, and evidently, have a direct impact on its metabolite composition (Figure 4B).

The higher concentration of fusel alcohols in the aged spirits provides evolving aromas and a fuller body of intense aromatic intensity.

The higher concentration of ethyl acetate in the aged spirits is due to the high porosity of French oak, which allows greater evaporation of water, and consequently favors the esterification reaction of acetic acid, whose product is ethyl acetate, and thus, its concentration increases during the aging process.

The increased concentration of saccharides stems from the degradation of hemicellulose in the barrel.

The enhanced concentration of aromatic metabolites further verifies that the aging process allows the inflow of ingredients from the oak shell of the barrel into the spirits, thereby influencing their composition. In particular, the thermal degradation of lignin enriches the aged distillates with metabolites bearing furanic and phenolic groups, which may exhibit antioxidant activity. These metabolites are mainly aldehydes (vanillin, sinapaldehyde) and phenolic acids (gallic acid, ellagic acid), which give the aged spirits a bright medium amber color depending on the wood environment in which it was matured. Furfural is the main metabolite of the furan group stemming from the interaction of the beverage with the wood.

Similar metabolites (syringaldehyde, coniferaldehyde) have been identified in the Italian grape pomace distillate grappa when aged in wooden barrels for at least one year [31]. This study also probed the importance of wood barrel type, i.e., oak or cherry in the final enrichment of the aged grappa, with the main compounds affected being coniferaldehyde, syringaldehyde (smoke/woody note), vanillin (vanilla), eugenol (clove), and guaiacol derivatives. Another attempt to characterize aged grape marc spirits from Spain (orujo) also revealed the migration of phenolics to the beverage in relation to the wood type [32].

Besides the aging procedure incorporating a wooden barrel, another approach uses the addition of oak chips. The impact on the volatile composition and sensory characteristics of Moravia Agria wines showed superior sensory attributes and increased levels of benzene compounds, oak lactones, and furanic compounds with the use of oak chips during MLF [33].

**Grape Marc Spirits from Peloponnesus:** To examine the effect of aging on multivariatal spirits (Moschofilero, Moscato, and Chardonnay) aged for two years in oak barrels, as well as on single-varietal Agiorgitiko aged for one year in oak barrels, a supervised analysis was also employed.

**Figure 5:**
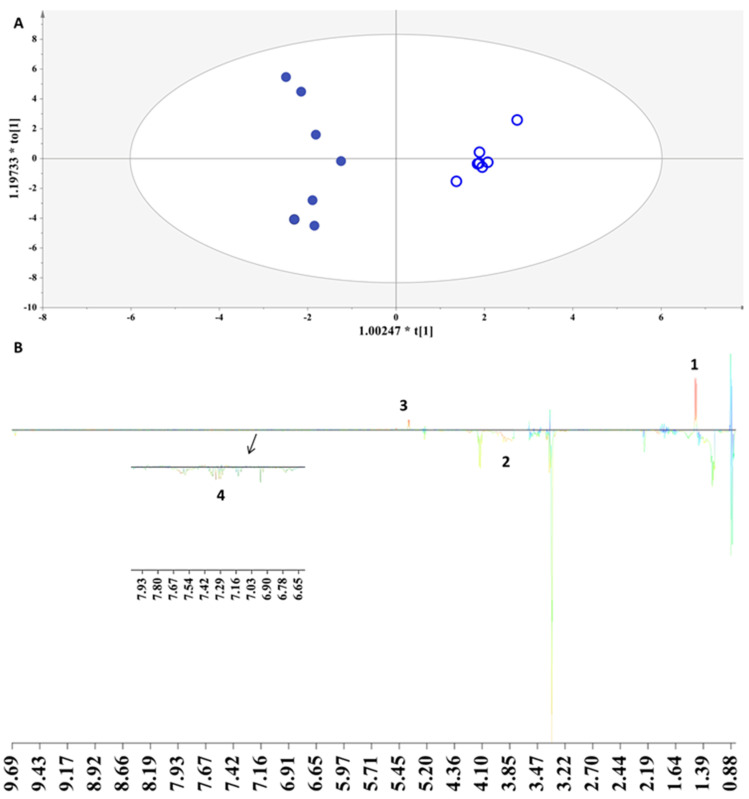
(**A**) OPLS-DA model on the multivarietal spirits (Moschofilero, Moscato, and Chardonnay) from Peloponnesus winery aged for two years in oak barrels. Samples representation coding: empty symbol = fresh grape marc spirit, full symbol = aged for two years in oak barrels grape marc spirit. Par scaling, A = 1 + 1, N = 14,R^2^x(cum) = 0.78, R^2^y(cum) = 0.96, Q^2^ (cum) = 0.92, *p*-value = 6.59528 × 10^−5^. (**B**) S-line plot (1,3:acetaldehyde hydrate, 2: saccharides (glucose, fructose, xylose, and arabinose), 4: phenolic and furanic metabolites).

In the OPLS-DA model (Figure 5), the multivarietal (Moschofilero and Moscato and Chardonnay) spirits were discriminated according to the first principal component into aged and fresh. The metabolites responsible for this discrimination are depicted in the corresponding S-Line plot (Figure 5B). Specifically, fresh spirits are characterized by acetaldehyde hydrate, while aged spirits are characterized by the presence of saccharides (glucose, fructose, xylose, and arabinose), as well as phenolic and furanic components.

**Figure 6:**
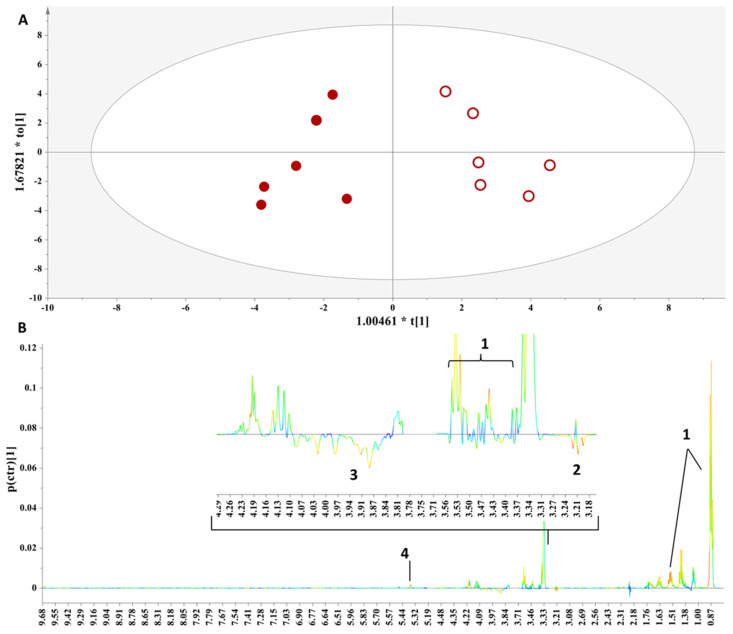
(**A**). OPLS-DA model on the single-varietal Agiorgitiko from Peloponnesus winery. Samples representation coding: empty symbol = fresh grape marc spirit, full symbol = aged for one year in oak barrels grape marc spirit. Par scaling, A = 1 + 1, N = 12, R^2^x(cum) = 0.86, R^2^y(cum) = 0.99, Q^2^ (cum) = 0.97, *p*-value = 0.022315. (**B**). S-line plot (1: fusel alcohols, 2: methyl acetate, 3: saccharides (glucose, fructose, xylose, and arabinose), 4: acetaldehyde hydrate).

In Figure 6A, the OPLS-DA model for Agiorgitiko spirits from Peloponnesus is displayed, clearly discriminating between the samples along the first principal component into aged and fresh spirits, and the corresponding S-line plot (Figure 6B) highlights the metabolites that contribute most to this discrimination. Aged spirits were characterized against fresh ones by the increased concentration of saccharides (glucose, fructose, xylose, and arabinose) and methyl acetate. On the other hand, the fresh grape marc spirits were related to increased content in acetaldehyde hydrate and fusel alcohols. In fact, the opposite trend was expected, as aged spirits feature stronger organoleptic characteristics. One explanation is that the one-year stay in the barrel was not enough to induce strong changes in their composition. A second interpretation is that barrels show reduced dynamics if they have been used several times in the aging process.

The S-line plot did not highlight the contribution of metabolites in the aromatic region. Most notably, only a year’s aging in the barrel did not benefit the beverage enough and did not allow sufficient time for the migration of several compounds from the wood to the beverage.

To resume, multivarietal (Moschofilero, Moscato, and Chardonnay) spirits from Peloponnesus aged for two years in oak barrels are shown with an enhanced saccharide and aromatic profile compared with the fresh ones. On the other hand, Agiorgitiko samples from Peloponnesus aged for one year in oak barrels benefit to a lesser degree, and this potentially implies that longer stays in the barrel are probably needed, or even blending with a metabolically richer variety.

### 3.3. Antioxidant Activity Profile

Furans and phenolics stemming from the oak barrel aging process were identified in the aged grape marc spirits with the employment of NMR metabolomics. The presence of such metabolites with a phenolic and/or furanic ring is probably linked to a potent antioxidant activity profile. Evidence already exists on other grape marc spirits from Spain or Italy that probes the relationship between the in vitro antioxidant properties and lignin-derived phenolics that are transferred from wooden barrels to wine spirits [3].

In this context, we attempted to achieve a comprehensive evaluation of the antioxidant activity profile of the samples using an array of well-established spectrophotometric assays (Folin–Ciocalteu, DPPH, ABTS•+, FRAP, CUPRAC, and the o-diphenols method). The different responses of the individual assays to evaluate the antioxidant capacity of food matrices have been reported in the literature [20,34,35]

The total phenolic and o-diphenolic content, as well as the free radical scavenging and reducing antioxidant capacities for the “fresh” grape marc spirits, was null. On the other hand, the assay results presented in Table 1 support, for the first time, evidence for the antioxidant capacity of the aged grape marc spirits. This fact aligns the antioxidant fingerprint with metabolites that derive from the wooden barrel and not from the raw material, the grape. This observation is very important, as it confers to the aged Greek grape marc spirits metabolites of enhanced added value compared with the “fresh” ones. The health benefits of moderate consumption of aged spirits can be attributed to their phenolic composition with enhanced bioactivity, such as free-radical scavenging, inhibiting lipid peroxidation, and reducing platelet aggregation and thrombosis, in opposition to ethanol-induced damage [3].

According to the mean values displayed in Table 1, the multivarietal spirits (Moschofilero, Moscato, and Chardonnay) aged in the oak barrel for two years feature higher phenolic and o-diphenols content and exhibit the most potent antioxidant activity profile, followed by the samples of Muscat Blanc aged for 1 year and the single-varietal Agiorgitiko, also aged for 1 year.

Also, a ranking of the spectrophotometric assays according to the values of the grape marc spirits can be detracted from Table 1. In fact, reducing antioxidant capacity assays exhibits higher values of antioxidant activity, whereas free radical scavenging activity assays lead to relatively much lower values. (The antioxidant capacity of the aged grape marc spirits was evaluated as (CUPRAC (106.0 ± 4.6) > FC (89.2 ± 3.8) > FRAP (78.0 ± 4.2) > OD (44.7 ± 3.4) > ABTS•+(31.1 ± 1.3) > DPPH (28.6 ± 1.2). It becomes evident that the application of more than one assay is deemed necessary to comprehensively assess the antioxidant activity profile of a grape marc spirit. The different responses of the methods may be attributed to the different phenolic, furanic, and diphenolic content of the aged grape marc spirit. The duration of aging and the type of barrel may also contribute to this variation [3].

Additionally, a correlation analysis of the measurements for the six different assays was implemented with the Pearson correlation method (Table 2). A high correlation was attributed between spectrophotometric methods with a common mechanism of action, i.e., free radical scavenging activity assays DPPH and ABTS•+, as well as reducing antioxidant capacity assays CUPRAC and FRAP. A strong correlation was observed between the Follin–Ciocalteu method and all the assays, whereas the o-diphenol content was moderately correlated with DPPH, ABTS•+, CURPAC, and FRAP. This hints at the presence of metabolites from various categories, such as furanic and phenolic metabolites, which contribute to the antioxidant potential and can be evaluated by both free radical scavenging activity as well as reducing antioxidant capacity assays.

The antioxidant activity profile of aged grape mark spirits translates into enhanced added value as compared with the fresh ones, also signifying that moderate consumption may confer a health benefit. This study underlines that phenolic aldehydes (such as sinapaldehyde, coniferaldehyde, and vanillin) and phenolic acids (such as vanillic acid and ellagic acid), previously identified in whiskey and now identified in Greek grape marc spirits, are significant antioxidants and may even contribute to the protection of blood vessels by triggering the activation of the *Heme Oxygenase 1* (HO-1) gene. Suzuki et al. probed metabolites in whiskey from wooden barrels that induced an increase in the cytoprotective HO-1 protein in human umbilical vein endothelial cells, potentially decreasing the risk of vascular diseases following moderate consumption, as opposed to freshly distilled whisky spirit, which exhibited no beneficial activity [36].

## 4. Conclusions

In summary, the NMR metabolomics protocol addressed the global metabolic profile of aged and fresh Greek grape marc spirits and attributed metabolic markers to their regional provenance, grape cultivar, and aging procedure.

The maturation in the barrel was generally associated with an increase in saccharides and an increase in the concentration of fusel alcohol, factors that contribute to the organoleptic properties of the aged spirits. The enrichment of the aromatic region was also highlighted with the identification of metabolites, with a furanic and phenolic ring derived, respectively, from the polysaccharides degradation or the thermal decomposition of lignin. Here, the multivarietal spirits (Moschofilero, Moscato, and Chardonnay) aged in the oak barrel for two years feature higher phenolic and o-diphenols content and exhibit the most potent antioxidant activity profile, followed by the samples of Muscat Blanc aged for 1 year and the single-varietal Agiorgitiko, also aged for 1 year. Their presence was linked to the appearance of antioxidant capacity in the Greek aged spirits, evidenced for the first time in aged *tsipouro* distillates by an array of spectrophotometric methods. In fact, a thorough assessment of the antioxidant potential of Greek aged grape marc spirits was attempted by applying complementary spectrophotometric methods, thus providing for the first time an accurate estimate of their “true” antioxidant properties. Thus, we highlighted that reducing antioxidant capacity assays exhibited higher values of antioxidant activity, whereas free radical scavenging activity assays led to relatively much lower values. The increased concentration of saccharides stems from the degradation of hemicellulose in the barrel. The enhanced concentration of aromatic metabolites further verifies that the aging process allows the inflow of ingredients from the oak shell of the barrel into the spirits, thereby influencing their composition.

Albeit, beyond the already applied methods, it would be useful if future research considered in vitro testing of lyophilized aged grape marc spirits samples on human cell lines against free radical formation induced by an oxidative agent. Such an approach could provide a clearer overview of the antioxidant effect of the studied substrate and its biological relevance.

The interaction with an oak cask enables the beverage to develop a metabolic bouquet of unique organoleptic characteristics and even offers health benefits when compared with freshly distilled grape mark spirits following moderate consumption.

## Figures and Tables

**Table 1:** A summary of the average results for the six spectrophotometric assays.

Assay	VR	VAG	ST
DPPH (mg GA/L)	33.6 ± 0.5 *	22.1 ± 0.1 *	29.5 ± 1.1 *
ABTS•+ (mg GA/L)	36.4 ± 0.6 *	24.1 ± 0.1 *	32.0 ± 1.1 *
CURPAC(mg GA/L)	121.7 ± 1.2 *^,a^	92.3 ± 4.6 *^,b^	101.4 ± 10.8 ^a,b^
FRAP (mg GA/L)	96.1 ± 4.3 *^,#^	66.4 ± 0.2 *^,c^	68.4 ± 7.3 ^#,c^
FC (mg GA/L)	105.9 ± 3.5 *	69.3 ± 0.6 *	89.5 ± 2.4 *
O-D (mg CA/L)	52.6 ± 2.8 *^,d^	30.9 ± 0.5 *^,$^	49.4 ± 7.9 ^d,$^

Samples coding as VR: two-year-aged multivarietal spirits (Moschofilero, Moscato, and Chardonnay) from Peloponnesus winery, VAG: one-year-aged single-varietal Agiorgitiko spirits from Peloponnesus winery, ST: one-year-aged single-varietal Muscat Blanc spirits from Thessaly. Each sample was measured in triplicates (Supplementary Material). The displayed values represent (mean ± SEM). Across each row, mean values are significantly different at the threshold, indicated as * *p* < 0.005, ^$^ *p* < 0.05, and ^#^ *p* < 0.01. Mean values with the same subscript letter are not significantly different. Mean values were compared via the two-tailed unpaired *t*-test.

**Table 2:** Correlation analysis of the six different methods.

N = 19	Pearson Correlation					
	DPPH	ABTS•+	CUPRAC	FRAP	FC	OD
DPPH	1.0					
ABTS•+	0.997 **	1.0				
CUPRAC	0.393	0.420	1.0			
FRAP	0.488 *	0.513 *	0.869 **	1.0		
FC	0.826 **	0.827 *	0.711 **	0.815 **	1.0	
OD	0.484 *	0.487 *	0.608 **	0.529 *	0.697 **	1.00

Statistical significance **. Correlation is significant at the 0.01 level (2-tailed). * Correlation is significant at the 0.05 level (2-tailed).

## Data Availability

The original contributions presented in the study are included in the article, further inquiries can be directed to the corresponding author.

## Supplementary Materials

The following supporting information can be downloaded at https://www.mdpi.com/article/10.3390/foods13111664/s1. Figure S1: A–G: Overlay of spectral regions for fresh (in red color) and aged (in green color) Grape Marc spirits with annotations on identified metabolites that are mainly present in the aged samples.; Figure S2: Validation of the OPLS-DA model in Figure 3, regarding two groups (100% Agiorgitiko, & 25% Moschofilero, 25% Chardonnay, 50% Muscat Hamburg), A. ROC Curves, B. Permutation testing; Figure S3: Validation of the OPLS-DA model in Figure 4, for samples from Thessaly discriminating fresh from aged samples, A. ROC Curves, B. Permutation testing; Figure S4: V alidation of the OPLS-DA model in Figure 5, on the multivarietal spirits (Moschofilero & Moschato & Chardonnay) that have remained in the oak barrel for two years discriminating fresh from aged samples, A. ROC Curves, B. Permutation testing; Figure S5: Validation of the OPLS-DA model in Figure 6, for samples from OPLS-DA model on the single-varietal Agiorgitiko that have remained in the oak barrel for one year discriminating fresh from aged samples, A. ROC Curves, B. Permutation testing; S6: A detailed description of the steps followed in each assay is presented; Table S1: Assignment of the furanic and phenolic metabolites including the resolved through ^1^D/^2^D NMR spectroscopy ^1^H και ^13^C resonances; Table S2: A summary of the results for the six spectrophotometric assays;
