# Supplementary material for: The Metabolic and Antioxidant Activity Profiles of Aged Greek Grape Marc Spirits
Source: Foods. 2024 May 26;13(11):1664. doi: 10.3390/foods13111664 (PMC11172063; doi:10.3390/foods13111664)
Supplement: Supplementary file 1 [file foods-13-01664-s001.zip › foods-2988666-supplementary.pdf]

The Metabolic and Antioxidant activity profiles of Aged Greek Grape marc spirits

Charalambos Fotakis[1†], Vasiliki Andreou[1], Dionysios C. Christodouleas[2] and Maria Zervou[1*]

SUPPLEMENTARY MATERIAL

Contents

Supplementary Fig. S1 A-G: Overlay of spectral regions for fresh (in red color) and aged (in green color) Grape Marc spirits with annotations on identified metabolites that are mainly present in the aged samples. 2

Supplementary Fig. S2: Validation of the OPLS-DA model in **Figure 3**, regarding two groups (100% Agiorgitiko, & 25% Moschofilero, 25% Chardonnay, 50% Muscat Hamburg), A. ROC Curves, B. Permutation testing. 6

Supplementary Fig. S3: Validation of the OPLS-DA model in **figure 4**, for samples from Thessaly discriminating fresh from aged samples, A. ROC Curves, B. Permutation testing. 7

Supplementary Fig. S4: Validation of the OPLS-DA model in **figure 5**, on the multivarietal spirits (Moschofilero & Moschato & Chardonnay) that have remained in the oak barrel for two years discriminating fresh from aged samples, A. ROC Curves, B. Permutation testing. 8

Supplementary Fig. S5 Validation of the OPLS-DA model in **figure 6**, for samples from OPLS-DA model on the single-varietal Agiorgitiko that have remained in the oak barrel for one year discriminating fresh from aged samples, A. ROC Curves, B. Permutation testing. 9

Supplementary Table S1: Assignment of the furanic and phenolic metabolites including the resolved through 1D/2D NMR spectroscopy ^{1}H και ^{13}C resonances. 10

Supplementary Table S2: A summary of the results for the six spectrophotometric assays. 12

Supplementary S6: A detailed description of the steps followed in each assay is presented 13

Supplementary Fig. S1 A-G: Overlay of spectral regions for fresh (in red color) and aged (in green color) Grape Marc spirits with annotations on identified metabolites that are mainly present in the aged samples.

The assignment of the rest of the resonance peaks (common in fresh and aged samples) is provided in Food Chemistry 138 (2013) 1837–1846; DOI: 10.1016/j.foodchem.2012.11.128

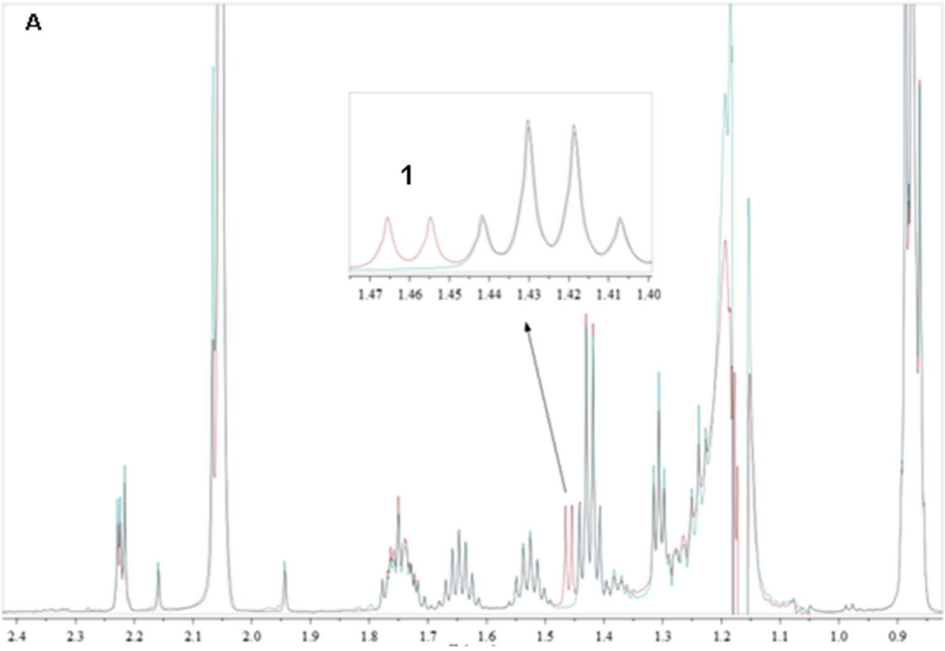

(1: 1-butanol)

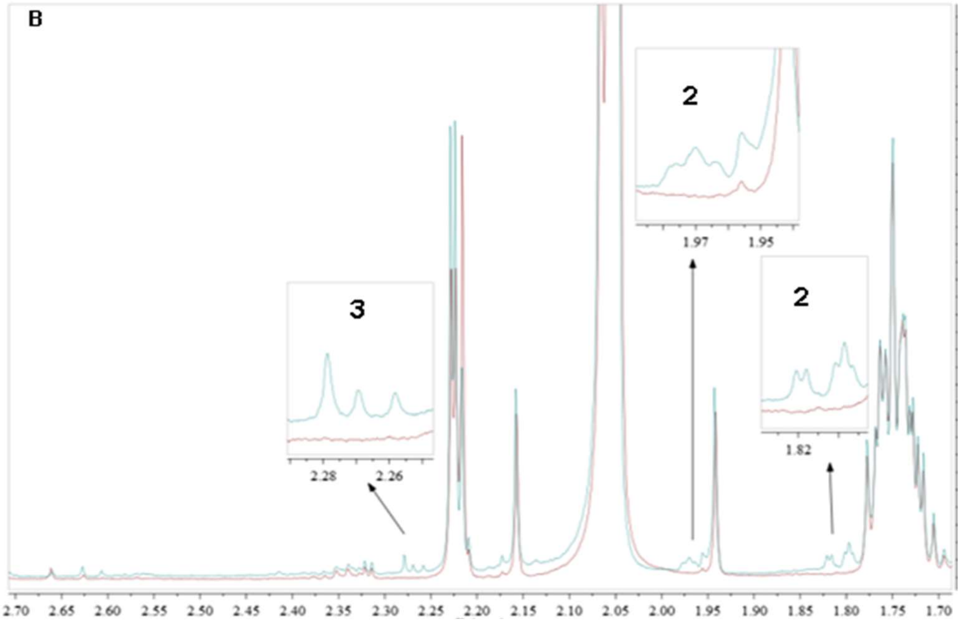

(2: proto-quercitol, 3: pyruvic acid)

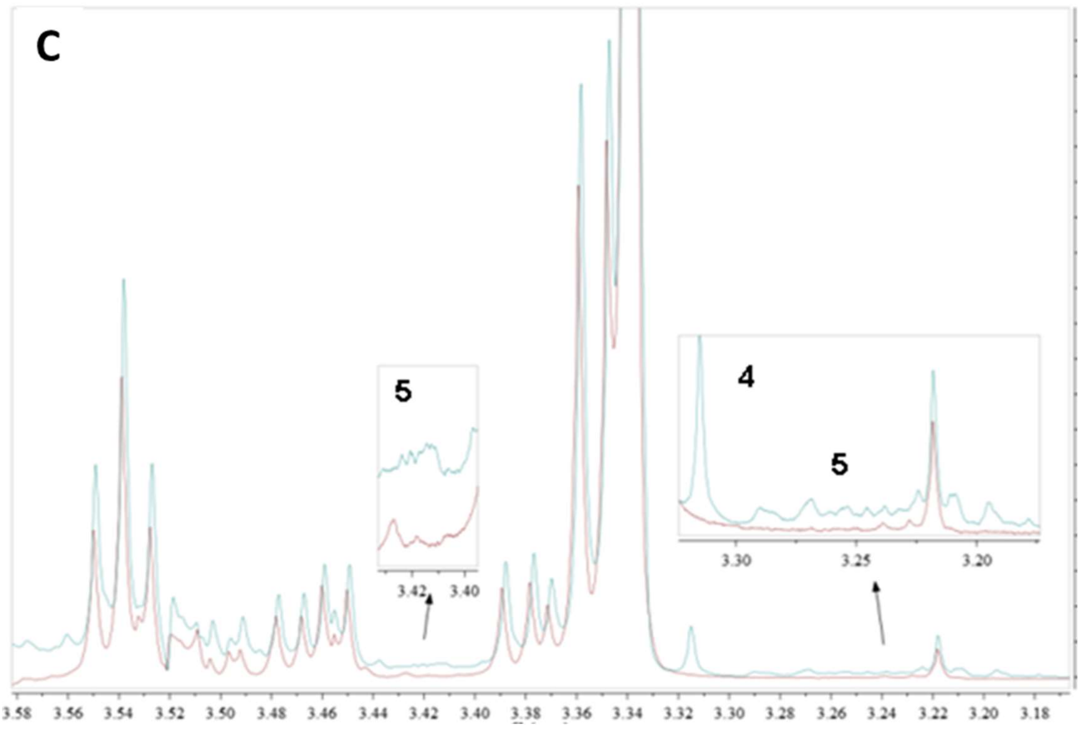

(4: Methyl acetate, 5: Glucose)

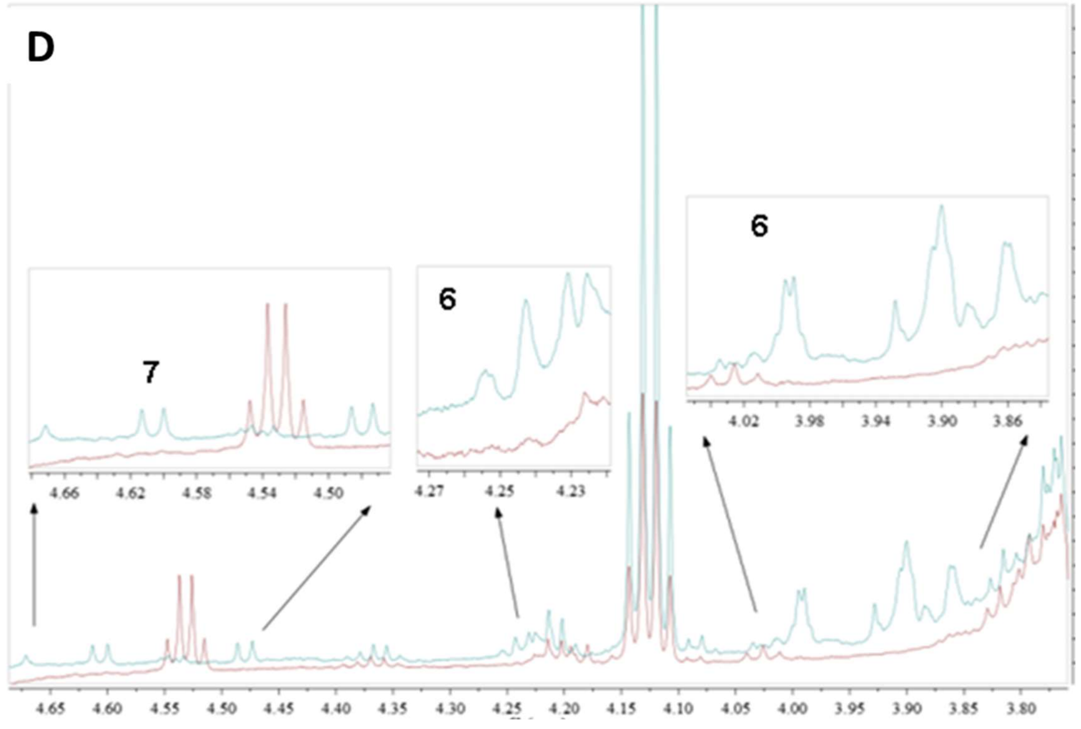

(6: Mono-saccharides, 7: Fructose)

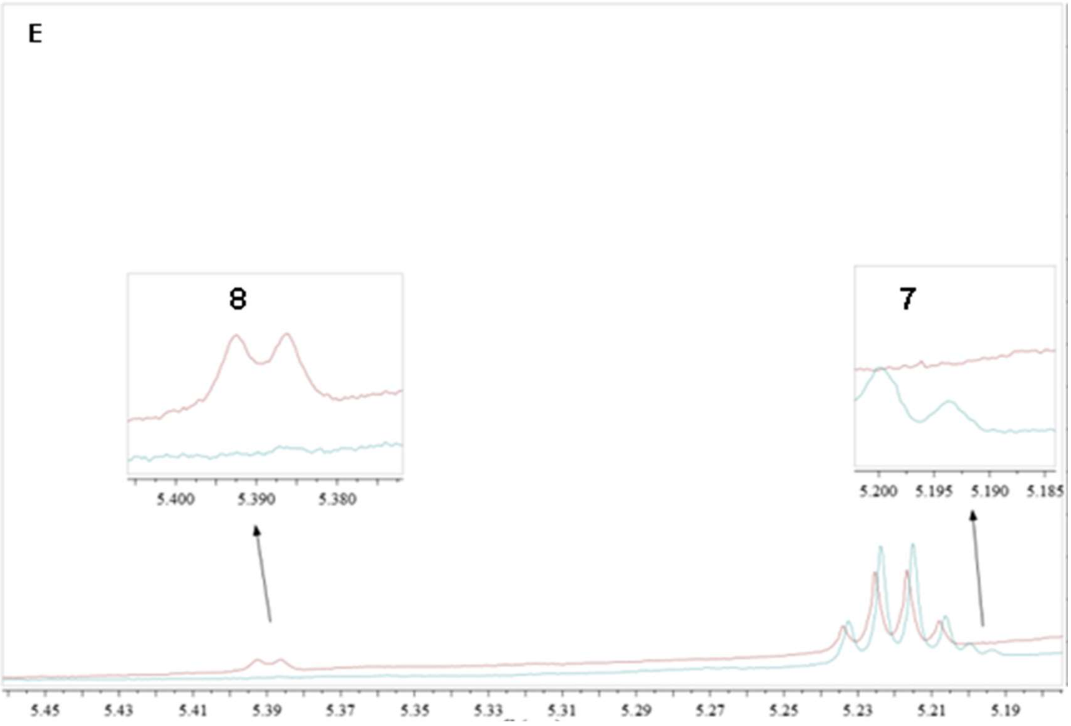

(7: Mono-saccharides, 8: Sucrose)

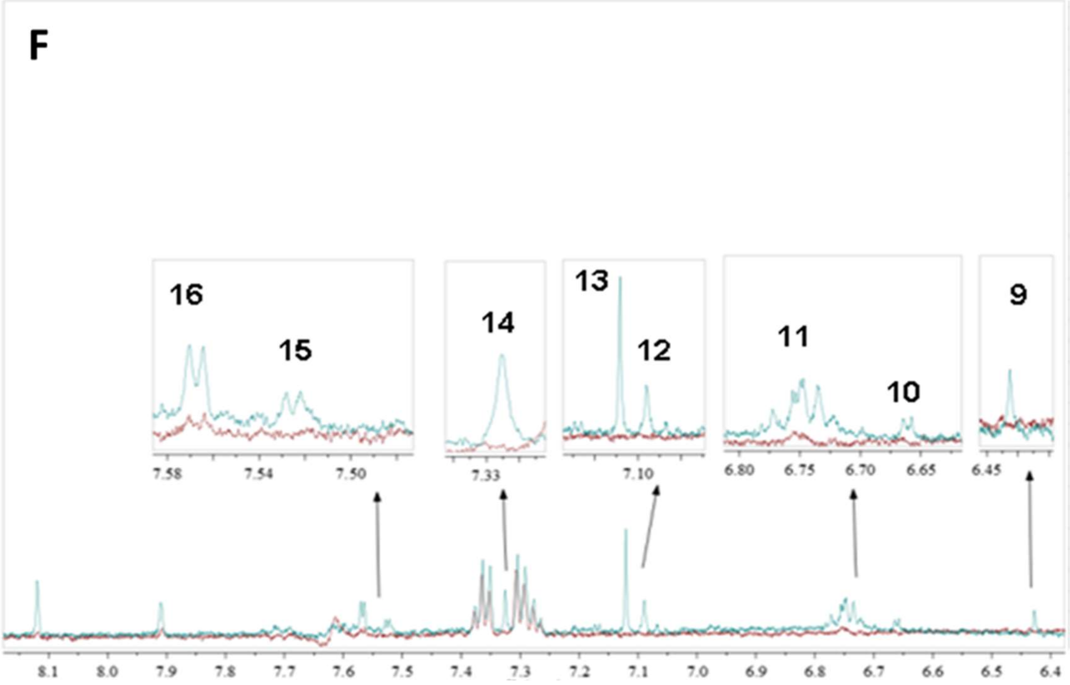

(9 & 10: sinapaldehyde, 11: acetovanillin & furfural & vanillin, 12: syringaldehyde, 13: gallic acid, 14: syringic acid, 15: furfural, 16: 5-methyl-furfural)

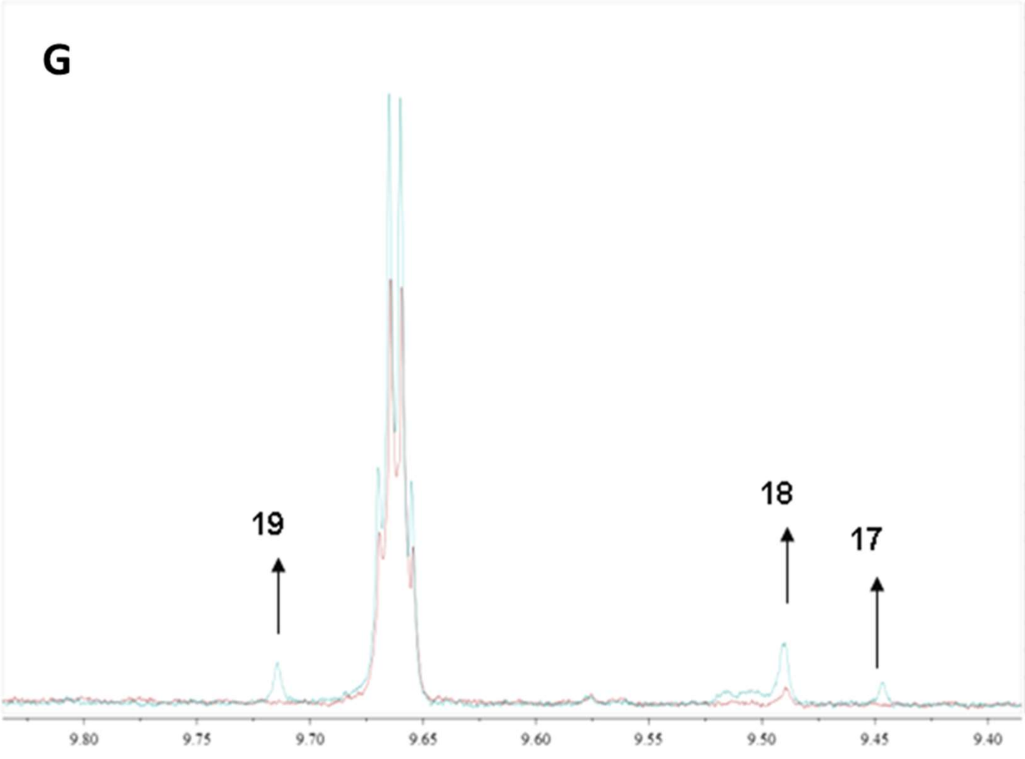

(17: Sinapaldehyde, 18: furfural, 19: 5-methyl-furfural)

Supplementary Fig. S2: Validation of the OPLS-DA model in **Figure 3**, regarding two groups (100% Agiorgitiko, & 25% Moschofilero, 25% Chardonnay, 50% Muscat Hamburg), A. ROC Curves, B. Permutation testing.

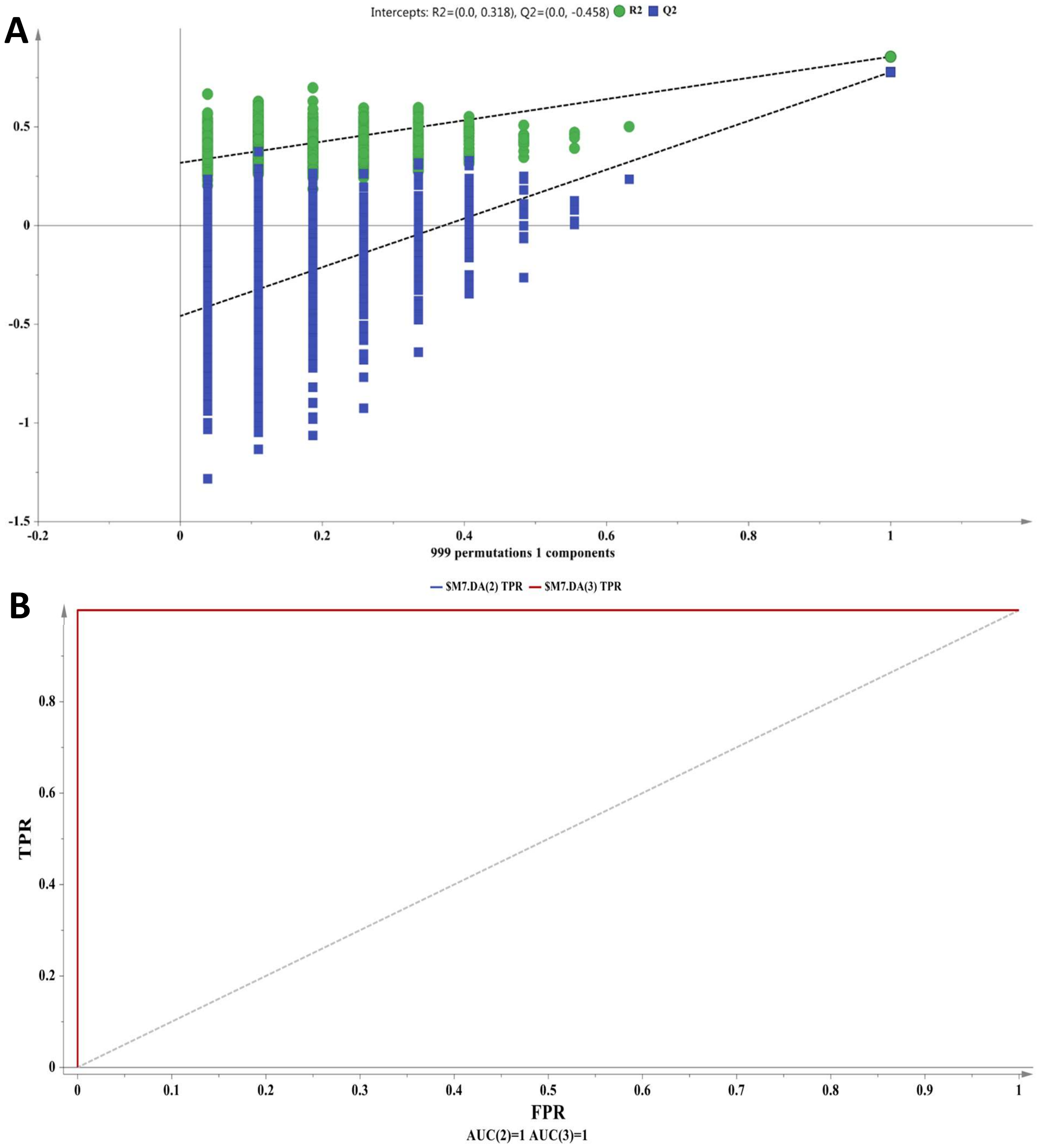

Supplementary Fig. S3: Validation of the OPLS-DA model in **figure 4**, for samples from Thessaly discriminating fresh from aged samples, A. ROC Curves, B. Permutation testing

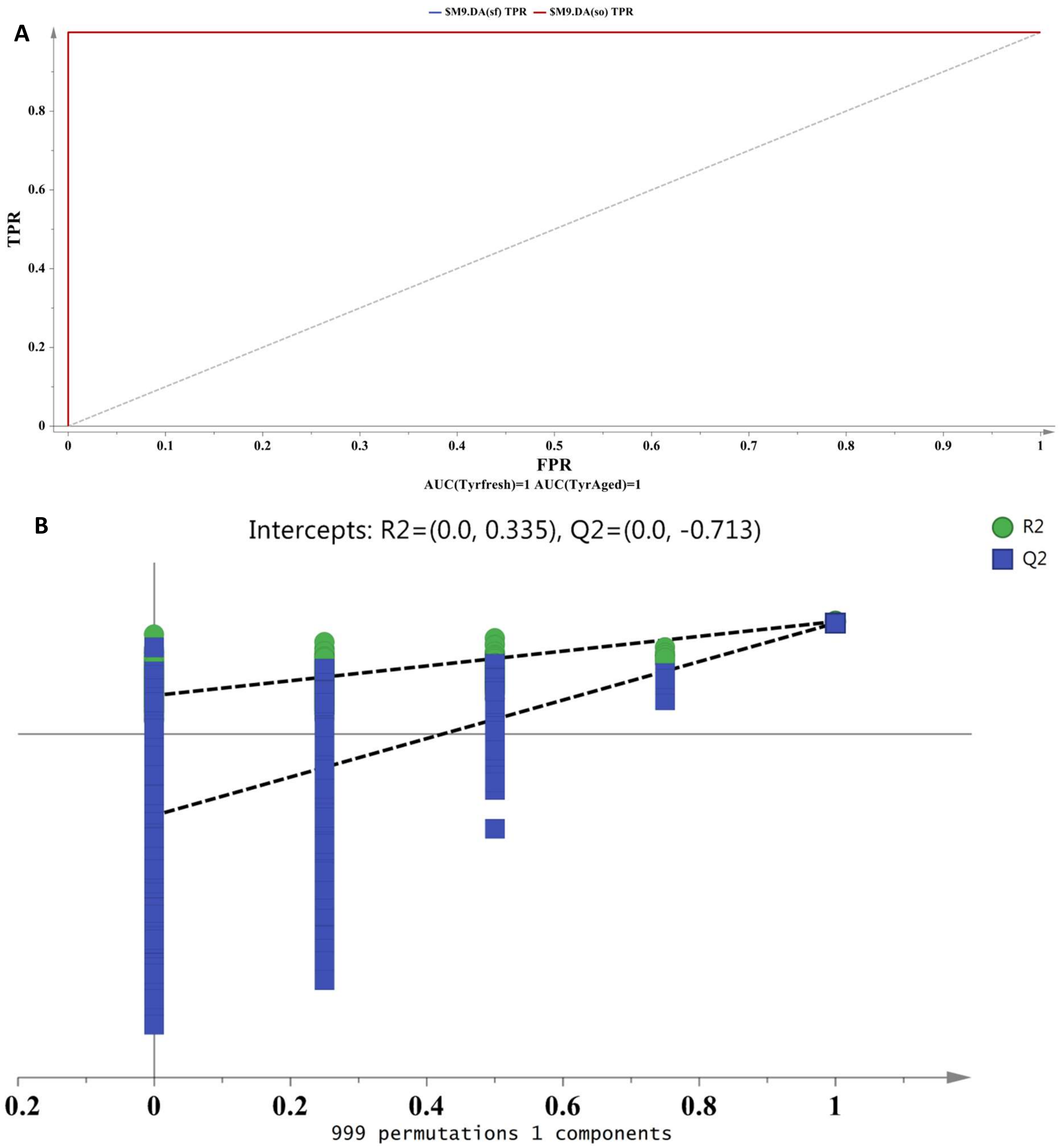

Supplementary Fig. S4: Validation of the OPLS-DA model in **figure 5**, on the multivarietal spirits (Moschofilero & Moschato & Chardonnay) that have remained in the oak barrel for two years discriminating fresh from aged samples, A. ROC Curves, B. Permutation testing.

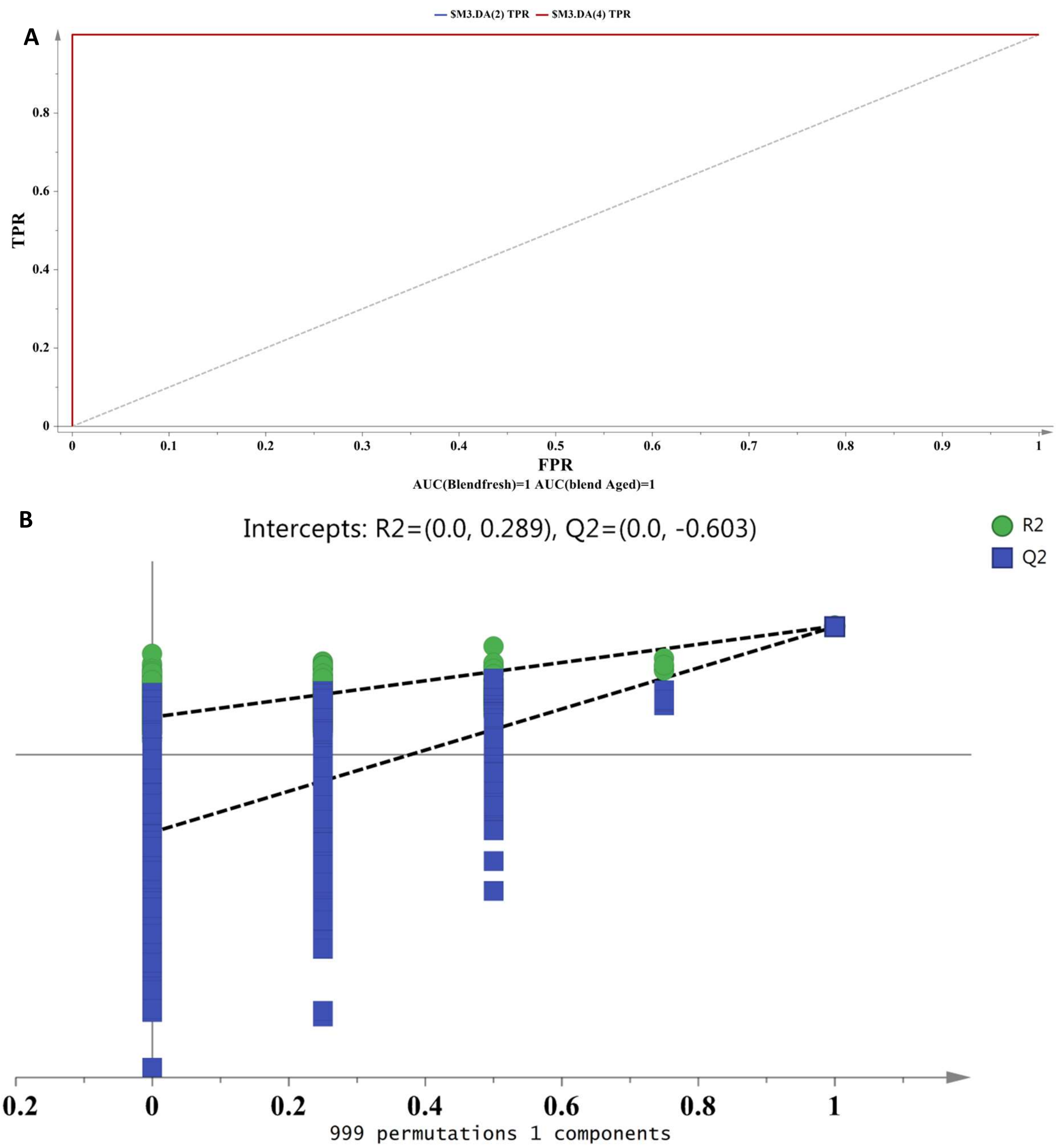

Supplementary Fig. S5 Validation of the OPLS-DA model in **figure 6**, for samples from OPLS-DA model on the single-varietal Agiorgitiko that have remained in the oak barrel for one year discriminating fresh from aged samples, A. ROC Curves, B. Permutation testing.

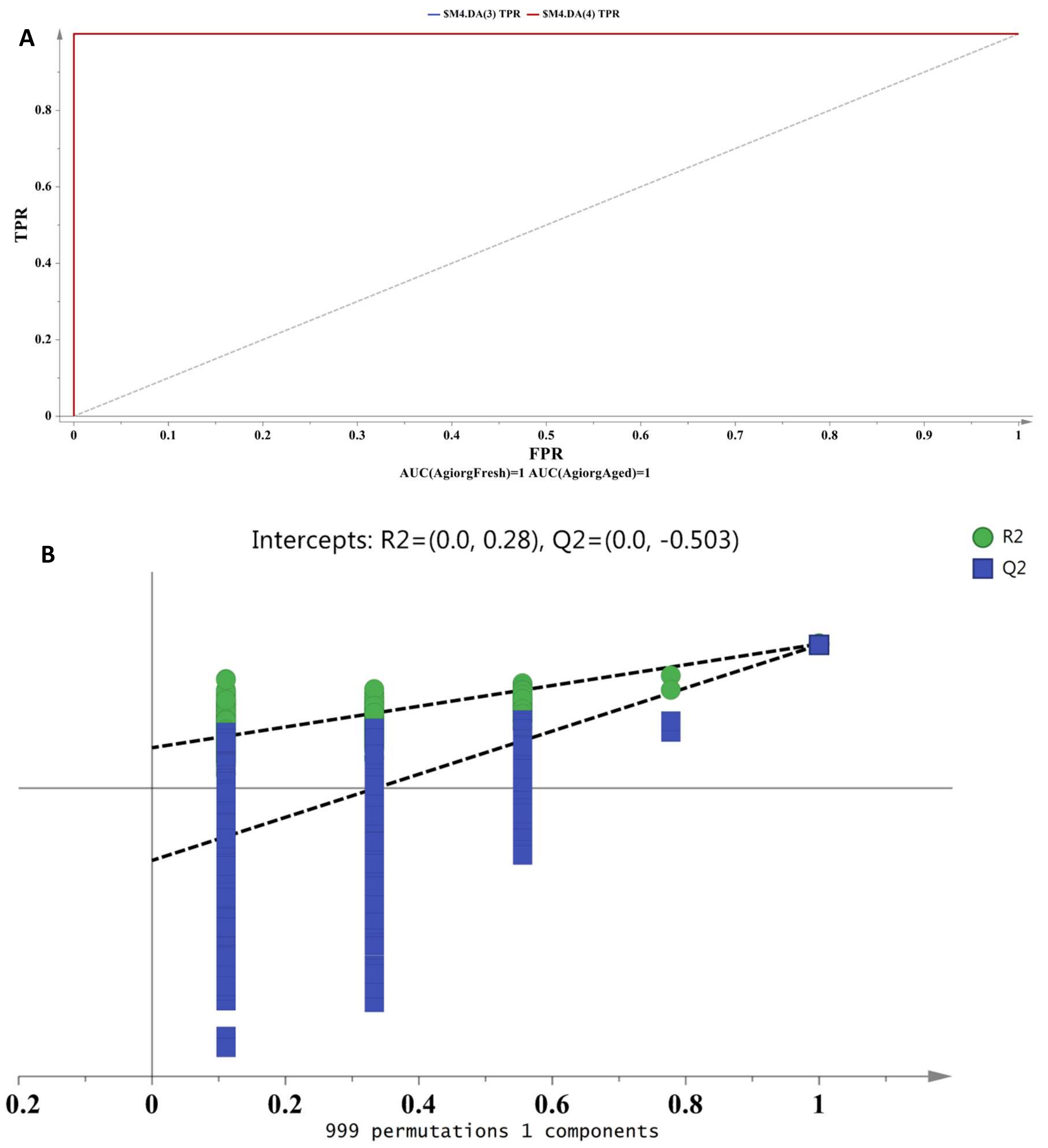

Supplementary Table S1: Assignment of the furanic and phenolic metabolites including the resolved through 1D/2D NMR spectroscopy ^{1}H και ^{13}C resonances.

	Chemical shifts of ^{1}H και ^{13}C		
		**metabolite**	**δ^{1}H/ δ^{13}C**
**1**	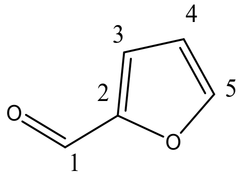	Furfural	**(1)**9.53 **(3)**7.38/129.64, **(4)**6.95/116.85, **(5)**7.95,
**2**	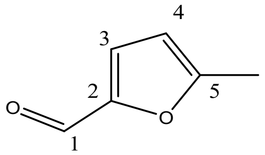	5-methylfurfural	**(1)**9.75, **(3)**7.54, **(4)**6.87,
**3**	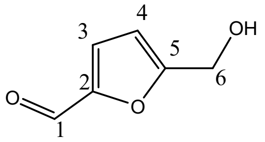	5-OH-methyl furfural	**(1)**9.68, **(3)**7.2, **(4)**6.34, **(6)**3.69,
**4**	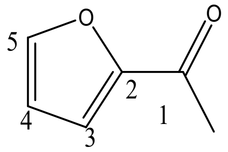	2-acetyl-furan	**(3)**7.19, **(4)**6.89, **(5)**7.26
**5**	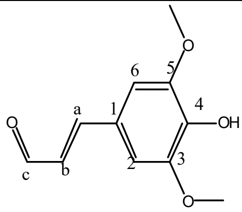	Sinapaldehyde	**(2,6)**6.37, (a)7.63, (b)6.67/106.25 (c)9.59
**6**	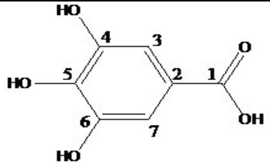	Gallic acid	**(3,7)**7.07/ 112.18
**7**	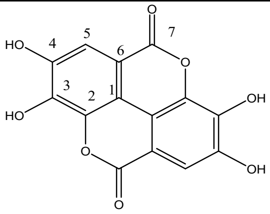	Ellagic acid	**(5)**7.84/112.75

**8**	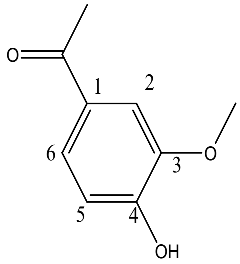	Acetovanillone	**(2,6)**7.21, **(5)** 6.87
**9**	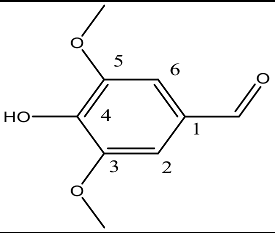	Syringaldehyde	**(2,6)**7.01/ 109.41
**10**	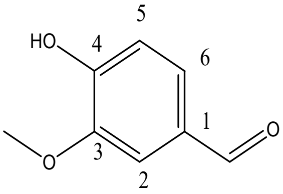	vanilline	**(5)**6.59, **(6)** 7.18
**11**	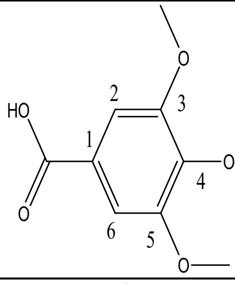	Syringic acid	**(2,6)**7.23/110.3, 3.9/ 51.18
**12**	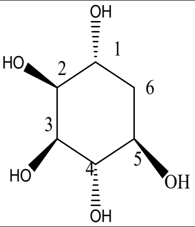	quercitol	(**1**)3.25/65.5, (**2**)3.33/78.6, (**3**)3.35/72.1 (**6**)1.87;1.92/32.9,
**13**	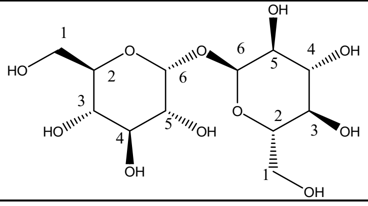	trehalose	**(1)5.17/95.50**
**14**	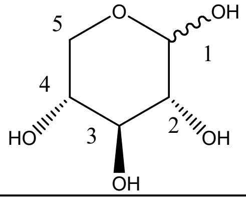	xylose	**(1)5.15/96.15, (3)3.75/72.02**

Supplementary Table S2: A summary of the results for the six spectrophotometric assays.

	DPPH	ABTS	CUPRAC	FRAP	FC	O-D
	(mg GA/L tsipouro) ± sd (n=3))					(mg CA/L) ± sd (n=3))
**VR.O1**	32.7 ± 0.21	35.7 ± 0.3	119 ± 3.0	89.8 ± 2.3	97.1 ±4.2	47.9 ± 3.1
**VR.O2**	32.1 ± 0.32	34.5 ± 0.2	122 ±4.0	93.6 ± 2.6	114 ±3.0	68.9 ± 2.5
**VR.O3**	32.5 ± 0.22	35.3 ± 0.2	120 ±4.0	91.1 ± 2.7	102 ±3.0	46.2 ± 2.2
**VR.O4**	33.6 ± 0.21	35.2 ± 0.3	123 ±5.0	96.1 ±2.9	113 ±3.0	52.6 ± 3.2
**VR.O5**	33.8 ± 0.44	36.9 ± 0.4	117 ±3.0	86.1 ±3.1	95.1 ± 4.3	51.7 ± 1.7
**VR.O6**	34.4 ± 0.33	37.7 ± 0.5	125 ±3.0	120.5 ±4.5	119.0 ± 2.0	50.8 ±2.4
**VR.O7**	35.8 ± 0.52	39.4 ± 0.7	126 ±4.0	95.2 ±4.2	101.0 ±3.0	50.0 ± 2.0
**VAG. O1**	22.3 ± 0.27	24.3 ± 0.3	100 ±4.0	66.6 ±3.2	68.2 ± 2.4	32.6 ± 2.4
**VAG. O2**	21.9 ± 0.13	23.8 ± 0.3	98.7 ±5.3	65.9 ± 3.6	67.4 ± 2.4	31.5 ± 1.7
**VAG. O3**	22.0 ± 0.14	24.1 ± 0.1	99.3 ±5.5	66.2 ±4.1	69.2 ± 2.6	31.4 ± 1.5
**VAG. O4**	22.1 ±0.22	24.2 ± 0.1	99.6 ±2.8	66.4 ±4.2	69.4 ± 3.2	30.6 ± 2.2
**VAG. O5**	22.0± 0.34	24.1 ± 0.2	74.5 ±2.3	66.2 ±3.7	70.3 ± 1.7	29.9 ± 2.1
**VAG. O6**	22.0 ±0.15	24.0 ± 0.3	81.8 ±4.4	67.3 ±3.8	71.3 ±1.5	29.3 ± 2.1
**ST.O1**	29.6 ± 0.33	32.3 ± 0.1	112 ±4.0	74.7± 2.8	89.3 ±2.3	33.7 ±1.9
**ST.O2**	28.0 ± 0.22	30.6 ± 0.3	112 ±3.0	75.4 ±2.9	92.1 ±3.1	41.9± 1.7
**ST.O3**	28.0 ± 0.22	30.4 ± 0.4	112 ±3.0	75.9 ±2.7	91.9 ±2,9	82.6 ± 2.8
**ST.O4**	28.1 ± 0.16	30.7 ± 0.21	112 ± 3.2	76.2± 2.5	92.3 ±3.4	50.2 ± 1.2
**ST.O5**	28.5 ± 0.16	31.1 ± 0.22	113 ± 3.3	76.1 ± 2.4	93.5 ± 2.8	58.4 ± 1.1
**ST.O6**	34,8 ±0.11	36,9 ± 0.10	47,2 ± 3.06	32, ± 3.81	77,8 ± 10.89	29,5 ± 2.85

Samples coding as VR: two years aged multivarietal spirits (Moschofilero & Moschato & Chardonnay) from Peloponessus winery, VAG: one year aged single-varietal Agiorgitiko spirits from Peloponessus winery, ST: one-year aged single varietal Muscat blanc spirits from Thessaly

Supplementary S6: A detailed description of the steps followed in each assay is presented

Determination of total phenolic content using the Folin-Ciocalteau assay

Aliquots of 1.0 mL of diluted grape marc spirit sample were mixed with 500 μL Folin-Ciocalteau Reagent. The mixture was left for 30 sec, then 4.0 mL of sodium carbonate (Na_2CO_3) 75 g/L was added and diluted with deionised water to total volume of 10 mL. The final solution was kept in the dark for 2 h and eventually the absorbance was measured at 765 nm[1]. The total phenolic content was expressed as mg gallic acid equivalents (GAE) per L of grape marc spirit, using a standard curve with 50–650 mg/L gallic acid ($y = 0.0017x - 0.0395$, $R^2 = 0.9920$).

Assessment of total O-diphenolic content

Equal volume of appropriate diluted (in deionised water) sample and sodium molybdate solution 5% w/v were mixed. The mixture reacted for 15 min and the absorbance was measured at 370 nm. As a standard substance aqueous solution of caffeic acid was used. Sodium molybdate solution 5% w/v was prepared using a mixture of 1:1 ethanol: deionized water as a solvent[2]. The results were expressed as caffeic acid equivalents (CAE) per liter of grape marc spirit.

Assessment of free radical scavenging activity using DPPH assay

200 μL of grape marc spirit sample were mixed with 3.2 mL of DPPH solution 0.1 mM. The absorbance was measured 10 min later at 525 nm. 2,2-Diphenyl-1-Picrylhydrazyl was diluted in a mixture of 2:1 CH_3CH_2OH:CH_3COONa 0.1 M and results were expressed as GAE per liter of grape marc spirit[3].

Assessment of free radical scavenging activity using ABTS•+ assay

200 μL of grape marc spirit sample, appropriately diluted with ethanol, was mixed with 5 mL ABTS•+ solution and 15 min later its absorbance was measured at 734 nm. ABTS•+ solution was prepared by mixing equal volumes of 7 mM aqueous solution of ABTS and 2.45 mM aqueous solution of potassium persulfate. The mixture was kept in the dark at 25 ^{0}C for 24 h and right before the measurement it was diluted with ethanol to adjust the absorbance at 0.700 (±0.02)[2]. Results were expressed as GAE per liter of grape marc spirit, as gallic acid was used as standard substance.

Assessment of reductive capacity using FRAP assay

An aliquot of 4.8 mL of FRAP reagent was mixed with 900 μL diluted grape marc spirit sample and left in darkness. After a time period of 15 min the absorbance of the mixture was measured at 593 nm. FRAP reagent consists of 25 mL buffer solution CH_3COOH-CH_3COONa 0.3 M (pH 3.6), 2.5 mL aqueous solution of $FeCl_3$ 2.5 mM, 20 mL TPTZ 10 mM, diluted in HCl 40 mM, and 3.0 mL deionised water[2]. Results were expressed GAE per liter of grape marc spirit.

Assessment of reductive capacity using CUPRAC assay

Equal volume of acetic ammonium buffer (1M) pH 7.0, $CuCl_2$ 10 mM, neocuproine 7.5 mM in ethanol and appropriate diluted (with ethanol) grape marc spirit sample, were added into a test tube. The mixture left for 30 min and then the absorbance was measured at 450nm[3]. The standard curve was made based on gallic acid as a standard substance. Results were expressed as GAE per liter of grape marc spirit.

---

[1] Andreou, V.; Strati, I.F.; Fotakis, C.; Liouni, M.; Zoumpoulakis, P.; Sinanoglou, V.J. Herbal distillates: A new era of grape marc distillates with enriched antioxidant profile. Food Chem. 2018, 253, 171–178

[2] Fotakis, C.; Christodouleas, D.; Zervou, M.; Papadopoulos, K.; Calokerinos, A.C. Classification of Wines Based on Different Antioxidant Responses to Spectrophotometric Analytical Methods. *Anal. Lett.* **2012**, *45*, 581–591, doi:10.1080/00032719.2011.649456

[3] Alañón, M.E.; Díaz-Maroto, M.C.; Díaz-Maroto, I.J.; Vila-Lameiro, P.; Pérez-Coello, M.S. Cyclic Polyalcohols: Fingerprints to Identify the Botanical Origin of Natural Woods Used in Wine Aging. *J. Agric. Food Chem.* **2011**, *59*, 1269–1274, doi:10.1021/jf104737n
